# Bilateral knee-load limiting during uneven-terrain walking induces hip-dominant load redistribution in predictive gait simulations

**DOI:** 10.3389/fmed.2026.1912600

**Published:** 2026-09-28

**Authors:** Heun-Jae Ryu, Dong-Kyun Koo

**Affiliations:** 1 Department of Physical Therapy, Honam University, Gwangju, Republic of Korea; 2 Department of Physical Therapy, Wonkwang University, Iksan-si, Republic of Korea

**Keywords:** gait biomechanics, joint-load redistribution, knee osteoarthritis, predictive neuromusculoskeletal simulation, uneven-terrain walking

## Abstract

**Background:**

Knee pain may induce protective gait patterns that reduce knee loading, but the multi-joint trade-offs that emerge under uneven-terrain demands remain uncertain.

**Methods:**

Using SCONE/Hyfydy, we generated sagittal-plane predictive simulations over a deterministic ten-tile terrain sequence. A reflex-based controller was optimized under no knee-load limit (C0) or bilateral soft limits of 4, 3, or 2 body weights (C4, C3, and C2). We retained the lowest-objective solution from 24 runs per condition and reanalyzed every complete stance within 1–12 s by limb and terrain.

**Results:**

The C0 knee joint-reaction-force peaks of 8.35/8.25 BW (left/right) occurred during early stance on tile 9 (−4°) and tile 10 (−2°), respectively. The left peak arose in a stance confined to tile 9, whereas the right peak arose in a stance that crossed the tile 9-to-10 boundary; neither occurred during initialization. Corresponding maxima were 4.51/4.34 BW in C4, 3.63/3.71 BW in C3, and 3.60/3.73 BW in C2. Mean stance impulse decreased from 2.14/2.00 BW·s in C0 to 1.79/1.82, 1.65/1.69, and 1.58/1.59 BW·s in C4, C3, and C2, respectively. Evaluation-window mean hip loading increased and speed decreased, whereas initial loading rate did not change monotonically. The selected solution ranked first by objective in every condition; 5–17 of 24 runs were within 10% of the selected objective.

**Conclusion:**

Within this model, controller, weighting scheme, and single terrain traversal, knee-load limiting was associated with slower walking, lower stance-phase knee peaks and impulses, and hip-dominant redistribution. These results are scenario-specific model behaviors rather than direct clinical predictions.

## 1 Introduction

Knee osteoarthritis (OA) is a leading contributor to pain-related disability in ageing populations. Contemporary global estimates indicate that OA prevalence and years lived with disability have increased substantially over recent decades, with the knee being among the most frequently affected joints and with a continuing upward trajectory expected as populations age and metabolic risk factors accumulate (1). Beyond chronic pain, knee OA is also clinically consequential because it compromises mobility and balance and is associated with an increased risk of falls, in part through impaired postural control and reduced neuromuscular reserve (2).

In daily life, walking rarely occurs on perfectly level ground. Even mild irregularities in terrain geometry can increase mechanical work requirements and muscle activation. Experimental evidence demonstrates that uneven-terrain walking increases mechanical work at the knee and hip and increases net metabolic energy expenditure relative to level walking, reflecting additional stabilization and propulsion demands (3).

Pain can induce rapid and sustained reorganization of motor control. Rather than a uniform reduction in movement, adaptation to pain is better understood as a redistribution of mechanical demands across tissues and tasks, reflecting a trade-off between protecting the painful structure and maintaining task performance (4). In human gait, this trade-off can manifest as altered joint kinematics, modified muscle recruitment, and load shifting toward adjacent joints (e.g., hip or ankle). However, experimentally isolating the causal influence of explicit knee load avoidance is challenging because pain, strength, confidence, and habitual compensation are deeply confounded in patient cohorts.

Predictive neuromusculoskeletal simulation provides a controlled in silico framework to test how movement strategies emerge from competing objectives and constraints (5). By optimizing controller parameters under explicit task goals, these simulations can generate dynamically consistent gait solutions without prescribing measured kinematics, although physiological credibility still depends on the model, controller, objective, and validation evidence. Recent work has shown that joint-load-avoidance objectives can reveal mechanistic trade-offs among loading, movement timing, and muscle recruitment (6). In contrast to that simulation of unilateral knee-pain-related standing-up, the present study applies bilateral soft knee-load limits during a deterministic multi-slope walking task and examines limb-, stance-, and terrain-resolved resultant joint-reaction-force outcomes and hip/ankle redistribution. This contribution is intended as a mechanistic scenario analysis rather than a patient-specific or level-ground causal estimate.

The objective of this study was to quantify how bilateral knee-load limiting, used as a simplified proxy for knee-load avoidance, shapes gait strategies during a deterministic uneven-terrain traversal in a sagittal-plane predictive simulation. We imposed progressively stricter soft thresholds on knee resultant joint reaction force (T = 4, 3, and 2 BW) and evaluated whether (i) stance-phase knee peaks and impulses and threshold-exceedance area were reduced, (ii) joint-load redistribution toward the hip and ankle emerged, (iii) sagittal pelvis and lower-limb kinematic adaptations accompanied unloading, and (iv) termination risk increased. We hypothesized that bilateral knee-load limiting would reduce knee loading and alter the distribution of mechanical demands across the lower limb.

## 2 Materials and methods

### 2.1 Study design

We performed predictive control optimizations under four conditions: a reference condition without a knee-load limit (C0) and bilateral soft knee-load-limit conditions of 4 BW (C4), 3 BW (C3), and 2 BW (C2). The number in each label denotes the imposed BW threshold. The same model, controller, terrain, and non-knee objective terms were used in every condition. The deterministic uneven terrain served as a repeatable stress test in which knee unloading, balance, and task performance competed. No separate level-ground baseline was simulated; therefore, this design did not estimate an independent effect of uneven versus level terrain. The simulation architecture and deterministic ten-tile sequence are illustrated in Figure 1. Conditions were optimized sequentially (C0 → C4 → C3 → C2), with the best solution from the preceding load-limited condition used to initialize the next stricter condition.

**Figure 1:**
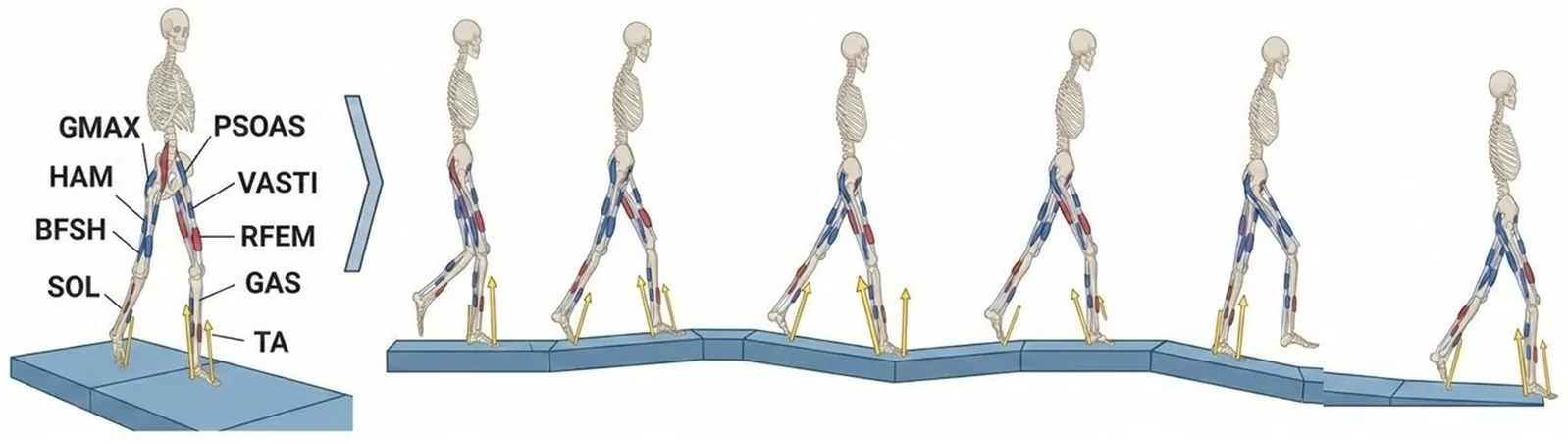
Overview of the neuromusculoskeletal simulation and deterministic ten-tile task. Tile 1 is an implicit level tile; tiles 2-10 use slopes 0°, +3°, 0°, −6°, +3°, 0°, −8°, −4°, and −2°. Arrows indicate ground reaction forces. Highlighted muscle groups are gluteus maximus (GMAX), iliopsoas (PSOAS), vasti (VASTI), rectus femoris (RFEM), hamstrings (HAM), biceps femoris short head (BFSH), gastrocnemius (GAS), soleus (SOL), and tibialis anterior (TA).

### 2.2 Simulation framework

Simulations and optimizations were executed in SCONE (Simulation-based Control Optimization of Neuromusculoskeletal Entities) (7) using the Hyfydy simulation engine (8). Optimization employed the Covariance Matrix Adaptation Evolution Strategy (CMA-ES) (9) within the SCONE pool-optimization framework to run multiple independent evolutionary searches in parallel.

### 2.3 Musculoskeletal model

We used the H0918v3 sagittal-plane neuromusculoskeletal model implemented in Hyfydy and distributed with the SCONE/Hyfydy example model set (7, 8). The nominal H0918 stature is 1.68 m, and the summed segment masses in the exact H0918v3 model file used here equal 74.5314 kg; BW normalization used this mass and gravitational acceleration. The model comprised nine degrees of freedom: horizontal and vertical pelvis translation, pelvis tilt, and bilateral hip, knee, and ankle flexion/extension. The head-arms-trunk assembly was one rigid segment attached to the pelvis, with no lumbar, thoracic, or metatarsophalangeal degree of freedom. Each leg was actuated by nine Hill-type musculotendon units (10), yielding 18 muscles in total. Muscle excitation-to-activation dynamics followed the OpenSim-style implementation in Hyfydy (8, 11).

Foot-ground contact was modeled using four spherical contact geometries (heel and toe spheres for each foot; radius 0.03 m) interacting with the terrain through a compliant Hunt-Crossley contact model with Coulomb friction (8, 12). The H0918v3 model file defined one abstract material named default_material for these contacts, with static and dynamic friction coefficients of 0.9 and 0.6, respectively; no empirical footwear-terrain material pair was specified. These coefficients were therefore modeling parameters inherited from the model configuration, not measurements assigned to a particular surface. The four-sphere configuration was retained as the minimal sagittal-plane heel/toe contact representation of the model and was kept identical across all conditions; no additional contact elements were introduced for the load-limiting conditions.

### 2.4 Uneven-terrain environment

Uneven terrain was generated with the SCONE/Hyfydy terrain blueprint. The fixed blueprint used random_seed = 2 and ten 1.0-m-long tiles. Tile 1 was an implicit initial level tile; the nine configured slope entries defined tiles 2–10 as 0°, +3°, 0°, −6°, +3°, 0°, −8°, −4°, and −2°, respectively. Thus, the ten-tile count and nine-entry slope list are consistent. Tile centers were located at x = 0, 1, .., 9 m, giving boundaries at x = −0.5, 0.5, .., 9.5 m. The same sequence was used in all conditions. Simulations lasted 12 s, and load outcomes were evaluated from 1 to 12 s. Terrain geometry was used as a reproducible challenge rather than as a set of repeated trials, and no terrain-sequence sensitivity analysis was performed.

### 2.5 Neuromuscular controller

Gait was generated using a state-based reflex controller (H0918RS2v3) organized into five gait states (EarlyStance, LateStance, Liftoff, Swing, and Landing). This controller structure follows established reflex-based walking control formulations (13). The controller combined muscle-level proprioceptive feedback (muscle length and force feedback) with load-based state transitions governed by a stance-load threshold, and included vestibular-like stabilization via proportional-derivative feedback on pelvis tilt acting on selected muscle groups. Neural transmission delays were included for hip-, knee-, ankle-, and vestibular-related feedback pathways (0.01–0.05 s, depending on pathway). Controller symmetry was enforced (symmetric = 1), meaning that optimized controller parameters were mirrored between the left and right limbs. Left-right differences in joint loads could still occur because each limb contacted different portions of the deterministic slope sequence at different gait-cycle times.

### 2.6 Objective function and soft-penalty terms

Each optimization minimized a composite objective comprising gait performance, effort, muscle activation, auxiliary ankle-position and knee-coordinate-force penalties, ground-reaction-force (GRF), and—only in C4-C2—bilateral knee-load terms. The common base objective was:

$$\begin{array}{rl} J_{base,c}= & 100J_{gait,c}+0.1J_{effort,c}+2000J_{activation,c}+0.1P_{ankle,c}\\ & +0.02P_{knee,c}+10J_{GRF,c} \end{array}.$$

$$J_{C0}=J_{base,C0}.$$

$$J_{c}=J_{base,c}+10\sum_{i\in \{L,R\}}\bar{\;p_{cap,i,c}},c\in \{C4,C3,C2\}.$$

Here, c ∈ {C0, C4, C3, C2}; T_c is 4, 3, and 2 BW for C4, C3, and C2, respectively; and the cap contribution is defined below. Jgait penalized step velocities below 0.8 m/s; Jeffort used the Wang2012 cost-of-transport formulation (14); Jactivation used average cubed activation; and JGRF penalized resultant GRF above 1.5 BW from 1 s onward. Pankle and Pknee denote the auxiliary ankle-position and knee-coordinate-force penalties, respectively.

The non-knee weights and auxiliary ±60° ankle penalty were inherited from the pre-existing H0918 scenario/measure configuration used to generate the baseline controller and were held fixed across conditions; the study-specific addition was the bilateral knee-load term. The H0918v3 model itself restricted ankle motion to −60° plantarflexion through +25° dorsiflexion, so the +60° auxiliary penalty did not permit 60° dorsiflexion and was nonbinding above the model's + 25° hard limit. No formal objective-weight sensitivity analysis was performed. Accordingly, the weights define one predictive scenario rather than a validated or unique physiological cost function.

### 2.7 Knee-load-limiting implementation

Knee-load avoidance was implemented as a bilateral soft penalty on the resultant internal joint reaction force at each modeled knee pin joint. The normalized force magnitude was:

$${\tilde{F}}_{knee,i}(t)=\frac{\|F_{knee,i}(t){\|}_{2}}{mg}=\frac{\sqrt{F_{x,i}^{2}(t)+F_{y,i}^{2}(t)+F_{z,i}^{2}(t)}}{mg}.$$

where i ∈ {L, R}, m is model body mass, and g is gravitational acceleration. Numerical reconstruction from the stored force components agreed with the exported knee.load variable within 1.2 × 10⁻⁵ BW. The reaction force arises from the multibody solution and therefore reflects the effects of muscle actuation, external contact, inertia, and gravity. It is not an axial tibiofemoral compressive force or a cartilage/contact estimate. Because the sagittal pin-joint model has no medial or lateral compartments, compartment forces were neither calculated nor combined.

For the load-limited conditions, the instantaneous penalty for each knee was:

$$p_{cap,i}(t;T_{c})=[max\{0,{\tilde{F}}_{knee,i}(t)-T_{c}\}{]}^{2}.$$

The scenario specified squared_penalty = 1, max = T_c, start_time = 1.0 s, and stop_time = 12 s. The mode attribute was omitted, so the SCONE default average mode was used. In continuous notation, the corresponding time-averaged penalty was:

$$\bar{\;p_{cap,i,c}}=\frac{1}{12\text{-}1}\int_{1}^{12}p_{cap,i,c}(t;T_{c})dt.$$

Each knee contributed with weight 10 to the composite objective.

The speed and knee-load requirements were implemented as soft penalties rather than hard constraints because the aim was to quantify how the optimizer traded knee unloading against task performance, effort, and stability, and because strict hard constraints can make predictive optimizations infeasible. Consequently, residual exceedance above the imposed knee-load threshold could remain in the optimized solutions; this residual exceedance was treated as an outcome rather than as a *post hoc* failure of the analysis.

### 2.8 Optimization protocol

For each condition, we executed 24 independent CMA-ES optimizations (pool size = 24) with up to 1,000 generations per run and a minimum progress criterion of 1 × 10⁻⁴. Up to eight optimizations were active and four ran concurrently. C4 was initialized from the baseline H0918GaitRS2Hfd4 parameter file, C3 from the best C4 solution, and C2 from the best C3 solution; C0 was optimized without a knee-load term. Controller updates used a 0.005-s step, and small random initial-state perturbations excluded pelvis translations and forward velocity. The lowest-total-objective trajectory was retained for biomechanical analysis.

Optimization diagnostics were computed from the lowest objective recorded in each of the 24 run directories. We report the selected minimum, median and range, and numbers within 10% and 25% of the selected score. Weighted objective-component contributions for the retained trajectories were reconstructed from stored per-frame penalty channels and configuration weights. Because filename-stored total objective values were rounded to three decimal places, the gait-component contribution was estimated as a residual and should be interpreted as approximate. Nonselected runs retained parameter snapshots but not replayed trajectories; therefore across-run similarity of gait strategies could not be evaluated.

### 2.9 Outcome measures and post-processing

For each retained solution, we quantified bilateral knee resultant joint reaction force, hip and ankle resultant joint reaction force, sagittal-plane kinematics, muscle activations, and early termination. Evaluation-window means were calculated from 1 to 12 s and include both stance and swing; they were retained only as descriptive summaries for the previously defined redistribution ratios and are not interpreted as weight-bearing contact metrics. Left and right values are reported separately before any bilateral ratio is formed. All force outcomes were normalized to BW.

A complete stance was defined from a Landing (state 4)-to-EarlyStance (state 0) transition through the final Liftoff sample before Swing (state 3). Only stances whose heel strike and stance end both occurred within 1–12 s were included. For each stance and limb, we calculated the maximum knee reaction force, trapezoidal force-time impulse, and average loading rate from heel strike to the stance maximum. Terrain at the maximum was assigned from center-of-pressure position using tile index = floor(xCOP + 0.5) + 1 for −0.5 ≤ xCOP < 9.5 m, consistent with the one-meter tiles centered at x = 0, 1, .., 9 m; positions outside this range were labeled pre- or post-terrain. The complete step-level dataset, including contacted tiles and timing, is provided as Supplementary Data S1.

Rotational coordinates were exported in radians and converted to degrees for tabular reporting. Raw hip_flexion retained its sign (positive = flexion), raw knee_angle was sign-reversed so positive values denote flexion, and ankle_angle retained its raw sign (negative = plantarflexion). Negative raw pelvis_tilt was reported as positive forward pelvis/whole-segment orientation. This variable does not represent lumbar motion because the model has no lumbar degree of freedom.

The redistribution index was defined as:

$$RI=\frac{F_{hip}+F_{ankle}}{F_{knee}}.$$

Knee exceedance time was the cumulative duration for which either knee exceeded the imposed threshold T over the 1–12 s evaluation window and was computed as:

$$D_{exceed}=\int_{1}^{12}I[max\{{\tilde{F}}_{knee,L}(t),{\tilde{F}}_{knee,R}(t)\}\;>T]dt.$$

Threshold-exceedance area was calculated as the sum of the left and right excesses:

$$A_{exceed}=\int_{1}^{12}({\left[{\tilde{F}}_{knee,L}(t)-T\right]}_{\;+\;}+{\left[{\tilde{F}}_{knee,R}(t)-T\right]}_{\;+\;})dt.$$

where [z]+ = max(z, 0). Thus, simultaneous left and right exceedance is counted once in duration but contributes each side's excess to the integrated area.

Step-normalized figures show ensemble means and within-simulation standard deviations across complete heel-strike-to-heel-strike cycles. Because these cycles span mechanically distinct tiles and conditions contain different cycle counts, the waveforms are descriptive visualizations rather than repeated-trial estimates of one homogeneous gait task. Exact, non-normalized stance outcomes are therefore reported by limb, stance, and terrain in Supplementary Data S1.

## 3 Results

All retained solutions completed the 12-s simulation. Across conditions, 63 complete stances fell wholly within the 1–12-s evaluation window (7–8 per limb and condition; Table 1 and Supplementary Data S1). Mean step velocity decreased from 1.15 m/s in C0 to 0.90–0.92 m/s in C4-C2, whereas step length remained 0.44–0.54 m. Because most terrain tiles were contacted once per limb, terrain-specific values are descriptive step outcomes rather than replicated estimates. Descriptive ensemble force profiles are shown in Figure 2.

**Table 1:** Spatiotemporal outcomes and joint-load summaries across conditions. Joint reaction forces are resultant pin-joint reactions normalized to body weight (BW), not compartmental contact forces. Evaluation-window means cover 1–12 s and include stance and swing. Stance begins at the Landing-to-EarlyStance transition and ends at entry into Swing; impulse is the trapezoidal integral over stance. Average loading rate is (stance maximum - heel-strike value)/time to maximum. Values are left/right (L/R). Exceedance time counts time when either knee exceeded T without double-counting simultaneous exceedance.

Metric	C0 (No limit)	C4 (T = 4 BW)	C3 (T = 3 BW)	C2 (T = 2 BW)
Spatiotemporal				
Step velocity (m/s)	1.15	0.90	0.92	0.90
Step length (m)	0.54	0.47	0.44	0.53
Joint reaction loads (BW; L/R)				
Knee evaluation-window mean	1.90/1.79	1.47/1.60	1.36/1.46	1.27/1.29
Knee stance-phase maximum	8.35/8.25	4.51/4.34	3.63/3.71	3.60/3.73
Hip mean	1.40/1.38	1.56/1.64	1.76/1.83	1.97/1.97
Hip peak	3.39/3.04	4.37/3.39	4.03/3.69	4.25/3.89
Ankle mean	1.99/1.94	1.86/1.95	1.80/1.91	1.81/1.87
Ankle peak	4.64/4.62	4.81/4.63	4.43/4.64	4.49/4.47
Knee-load threshold exceedance				
Knee exceed time (s)	−	0.24	0.49	5.97
Knee exceed area (BW·s)	−	0.05	0.11	1.64
Redistribution				
Redistribution index ((Hip + Ankle)/Knee)	1.80	2.28	2.58	2.98
Stance-specific knee JRF outcomes				
Complete stances (n) L/R	8/8	8/8	8/8	7/8
Mean stance peak (BW) L/R	4.88/4.23	3.45/3.37	2.96/3.02	2.71/2.80
Mean stance impulse (BW·s) L/R	2.14/2.00	1.79/1.82	1.65/1.69	1.58/1.59
Mean average loading rate (BW/s) L/R	30.03/17.20	35.80/24.28	17.40/23.01	28.68/28.41

**Figure 2:**
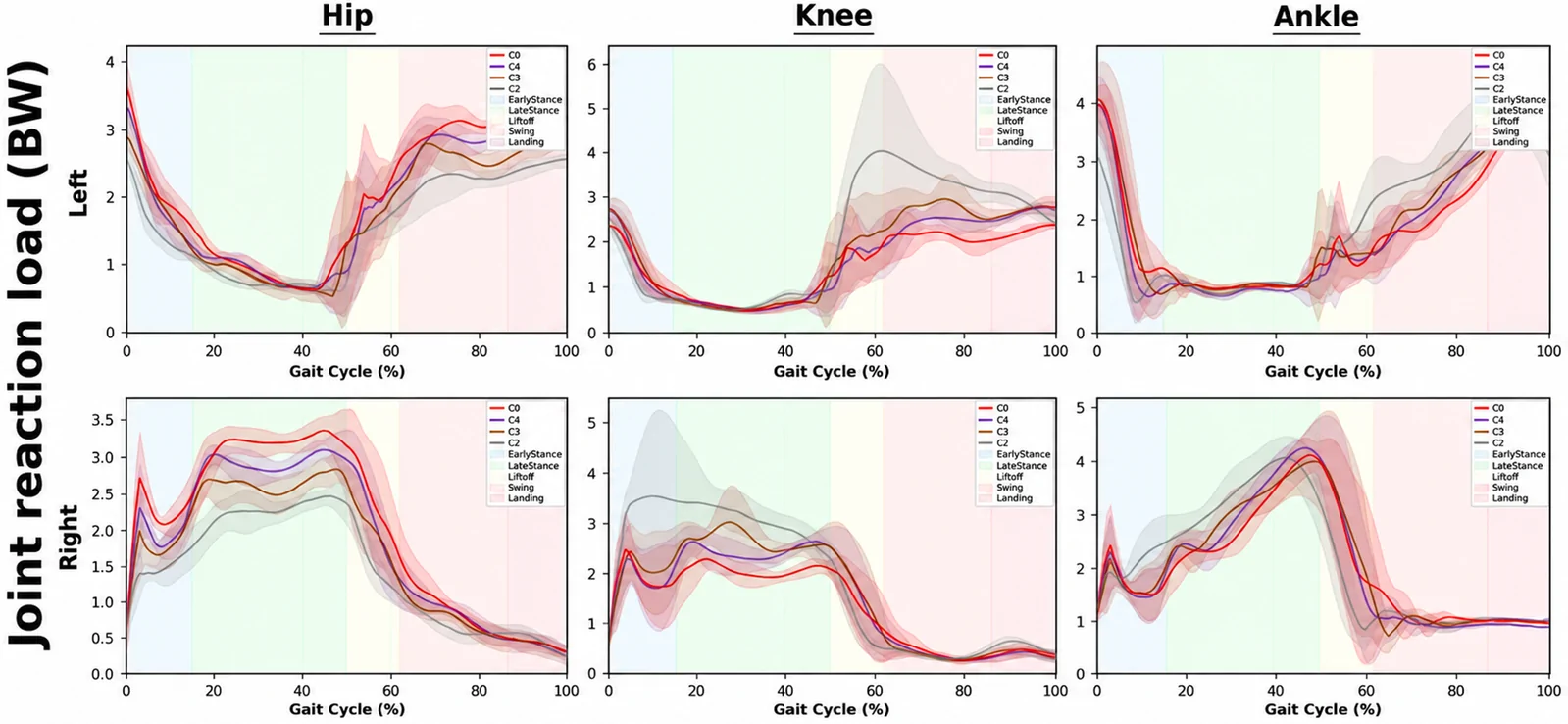
Ensemble hip, knee, and ankle resultant joint-reaction-force profiles across complete steps. Forces are normalized to BW and separated by limb. Solid lines and bands are within-simulation means and SDs across mechanically distinct steps and are descriptive rather than repeated-trial estimates of a homogeneous gait task. Exact stance- and terrain-specific knee outcomes are in Supplementary Data S1. Conditions: C0, C4, C3, and C2.

### 3.1 Task performance and knee-load limiting

The C0 maxima were localized to specific downhill stances. The left peak of 8.35 BW occurred at 6.60 s during early stance of the sixth full-simulation left stance (fifth complete stance in the analysis window) on tile 9 (−4°), and that stance contacted only tile 9. The right peak of 8.25 BW occurred at 7.24 s during early stance of the sixth right stance (fifth analyzed stance) on tile 10 (−2°). Its peak center of pressure was x = 8.547 m, 0.047 m beyond the tile 9-to-10 boundary at x = 8.5 m, and the stance crossed from tile 9 (−4°) to tile 10 (−2°). Neither peak occurred during initialization or a post-terrain step; the left peak was not a transition event, whereas the right peak occurred within a transition stance. Load-limited maxima were 4.51/4.34 BW in C4, 3.63/3.71 BW in C3, and 3.60/3.73 BW in C2 (left/right). Their peak tiles were −2°/−8° in C4, −4°/−2° in C3, and −4°/−2° in C2 (Supplementary Table S1). Thus, the extreme C0 values were associated with two different downhill-task contexts, and maxima under load limiting were not confined to the steepest decline.

Across complete stances, mean stance peak knee reaction force was 4.88/4.23 BW in C0, 3.45/3.37 BW in C4, 2.96/3.02 BW in C3, and 2.71/2.80 BW in C2. Mean stance impulse decreased from 2.14/2.00 BW·s in C0 to 1.79/1.82, 1.65/1.69, and 1.58/1.59 BW·s in C4, C3, and C2. Bilateral-average impulse reductions relative to C0 were 12.6%, 19.3%, and 23.3%, respectively. Average loading rate from heel strike to the stance maximum did not change monotonically (Table 1), indicating that peak and impulse reductions did not guarantee a slower initial rise. Threshold exceedance time/area remained 0.24 s/0.05 BW·s in C4, 0.49 s/0.11 BW·s in C3, and 5.97 s/1.64 BW·s in C2. Stance-specific summary values are provided in Supplementary Table S2, with complete records in Supplementary Data S1.

### 3.2 Joint-load redistribution

Evaluation-window mean hip load increased from 1.40/1.38 BW (C0) to 1.56/1.64 BW (C4), 1.76/1.83 BW (C3), and 1.97/1.97 BW (C2), whereas mean ankle load changed modestly. Consequently, the descriptive redistribution index ((hip + ankle)/knee) increased from 1.80 to 2.98. These 1–12-s means include stance and swing and should be interpreted only as time-averaged inter-joint allocation within each simulation, not as clinical contact-load exposure.

Hip peak loads increased relative to C0, whereas ankle peak loads remained similar (Table 1). The stance-specific knee results above show that the redistribution pattern was accompanied by lower weight-bearing knee impulse, not only by lower whole-window means.

### 3.3 Kinematic and muscle-activation adaptations

Kinematic differences were smaller than kinetic differences (right: Figure 3; left: Supplementary Figure 1). The reported pelvis-derived forward orientation varied by approximately 1–2° in mean value across conditions, while its range of motion decreased from 21.8° in C0 to 17.6°, 14.7°, and 10.5° in C4, C3, and C2 (Table 2). This coordinate describes pelvis/rigid-segment orientation and cannot be used to infer lumbar motion.

**Figure 3:**
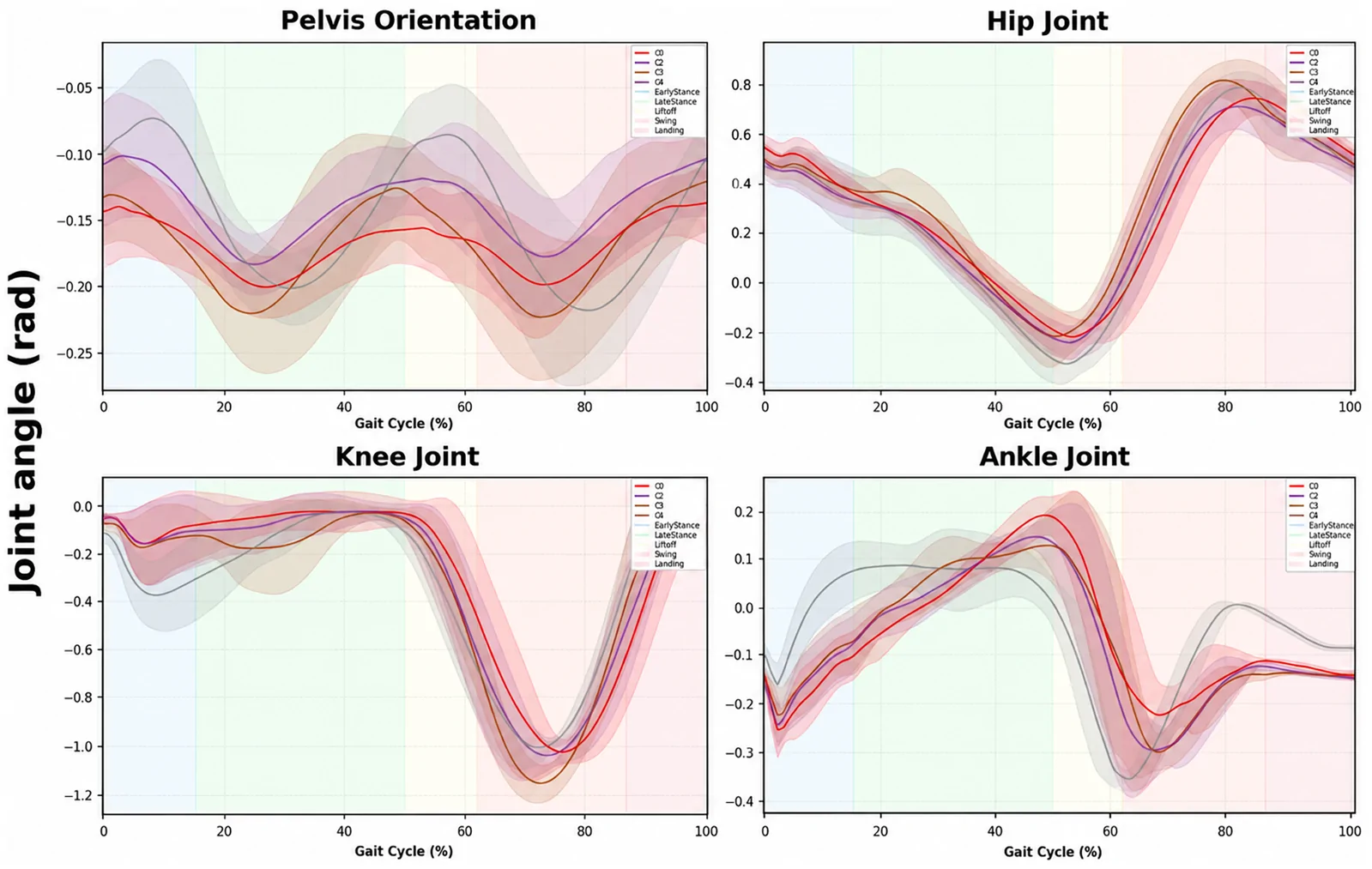
Sagittal-plane kinematic trajectories for the right side. Raw SCONE radian coordinates are shown without sign reversal. Hip_flexion is positive for flexion, knee_angle becomes more negative with flexion, and ankle_angle becomes more negative with plantarflexion. Degree-converted summaries are in Table 2. Bands show within-simulation SD across mechanically distinct steps. Conditions: C0, C4, C3, and C2.

**Table 2:** Condition-wise kinematic summary (evaluation-window mean and range of motion, ROM). Radians were converted to degrees. Knee flexion is positive after sign reversal of raw knee_angle; ankle angle retains the raw sign (negative = plantarflexion). Pelvis forward tilt is the sign-reversed global pelvis_tilt coordinate and does not represent lumbar motion. Bilateral values are L/R.

Metric	C0 (No limit)	C4 (T = 4 BW)	C3 (T = 3 BW)	C2 (T = 2 BW)
Pelvis orientation				
Pelvis forward tilt mean (deg)	8.6	9.5	8.1	9.8
Pelvis forward tilt ROM (deg)	21.8	17.6	14.7	10.5
Lower-limb joints (L/R)				
Hip flexion mean (deg)	15.7/14.8	19.4/19.0	15.6/15.2	16.5/16.4
Hip flexion ROM (deg)	77.5/83.3	86.4/86.9	78.2/70.5	79.5/71.8
Knee flexion mean (deg)	20.2/20.4	20.4/20.0	19.4/17.9	17.5/18.2
Knee flexion ROM (deg)	72.9/66.5	73.8/76.0	68.2/69.1	68.4/69.0
Ankle angle mean (deg)	−1.7/−1.8	−3.7/−3.1	−4.1/−3.6	−3.2/−2.8
Ankle angle ROM (deg)	46.6/35.6	48.5/36.2	42.1/34.5	45.1/37.6

Mean knee flexion decreased slightly in the stricter limit conditions (average across sides: 20.3 degrees in C0 vs. 18.6 degrees in C3 and 17.9 degrees in C2), indicating a more extended knee posture, while mean hip flexion and ankle angles remained in similar ranges (hip flexion: 15–19 degrees; ankle angle: −1.8 to −3.9 degrees; Table 2).

The muscle activation profiles were consistent with the hip-dominant redistribution observed in the joint-load outcomes (right: Figure 4; left: Supplementary Figure 2). Average vasti activation decreased from 0.12 in C0 to 0.08 (C4), 0.06 (C3), and 0.05 (C2), with a concomitant reduction in peak vasti activation from approximately 1.00 in C0 to 0.43–0.51 in C4, C3, and C2. In contrast, mean gluteus maximus activation increased from 0.04 (C0) to 0.12 (C4), 0.20 (C3), and 0.24 (C2), and peak gluteus maximus activation increased from 0.15 to 0.35–0.52.

**Figure 4:**
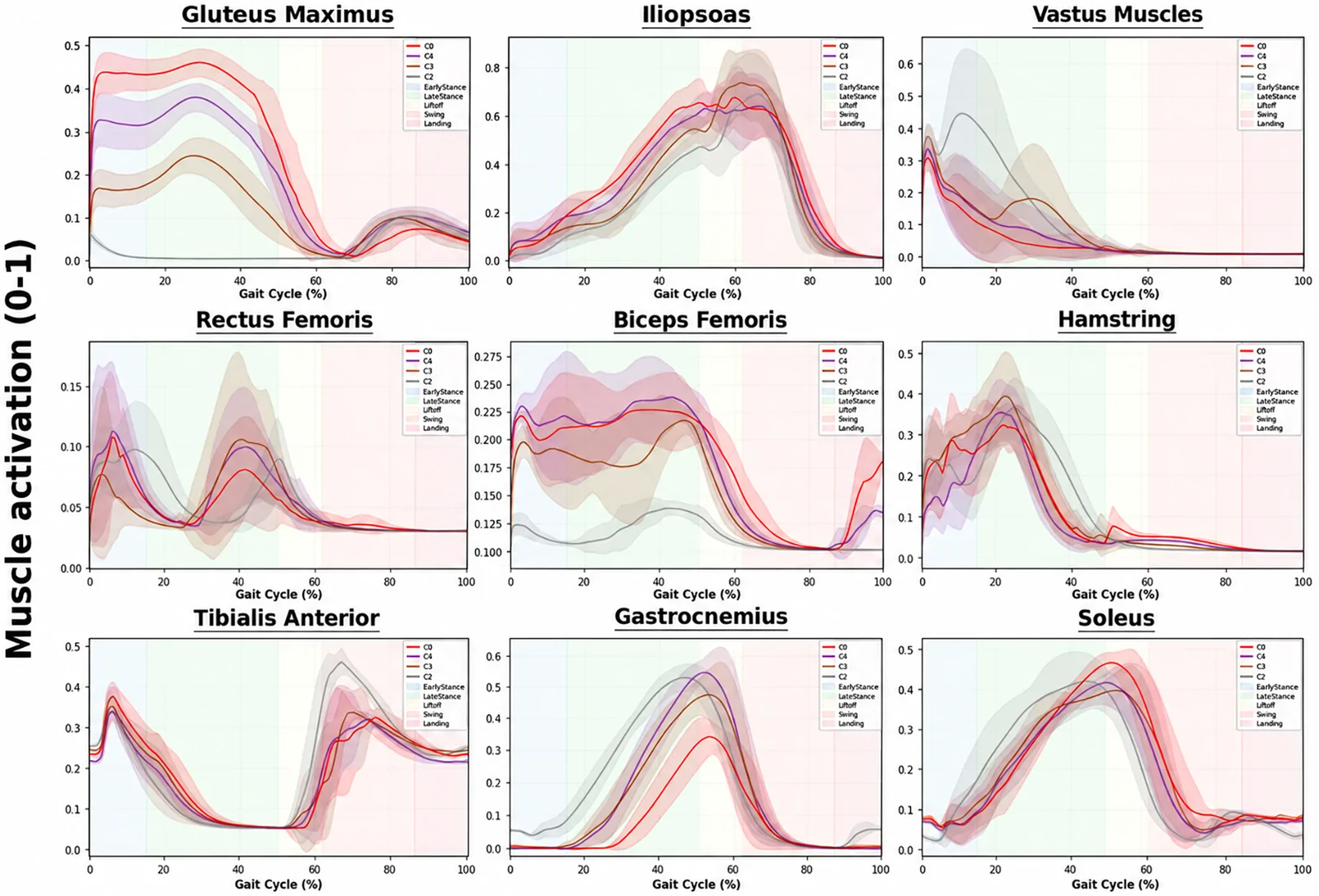
Muscle activation profiles (0–1, unitless) across the gait cycle for the right side under the reference and knee-load-limited conditions. Muscles shown are gluteus maximus, iliopsoas, vasti, rectus femoris, biceps femoris, hamstrings, tibialis anterior, gastrocnemius, and soleus. Solid lines indicate mean activation and shaded bands indicate +/−SD across steps within the uneven-terrain sequence. Background shading denotes gait subphases. Conditions: C0, C4, C3, and C2.

Iliopsoas activation also increased (mean: 0.24 in C0 vs. 0.29–0.34 in C4, C3, and C2). Gastrocnemius activation decreased (mean: 0.22 in C0 to 0.10–0.17 in C4, C3, and C2), whereas soleus and tibialis anterior showed smaller changes across conditions.

The selected solution was the minimum-objective run in each condition. Across the 24 runs, median (range) objectives were 1.657 (1.470–1.991) for C0, 2.276 (1.778–7.146) for C4, 2.144 (1.901–2.913) for C3, and 3.124 (2.866–3.372) for C2. The numbers within 10% of the selected objective were 6, 5, 8, and 17, respectively (Supplementary Table S3). Weighted component reconstruction showed that muscle activation was the largest component in every selected solution (0.926–1.160); the knee-cap component was 0.011 in C4, 0.037 in C3, and 0.771 in C2 (Supplementary Table S4). These score distributions show that the minima were not isolated objective outliers, but do not establish biomechanical representativeness because nonselected trajectories were unavailable.

## 4 Discussion

### 4.1 Principal findings and biomechanical trade-offs

In the retained lowest-objective solutions, soft bilateral knee-load limiting reduced the bilateral-average maximum stance reaction force by 46.7% in C4 and 55.8% in C3 and C2 relative to C0, while mean stance impulse fell by 12.6%, 19.3%, and 23.3%. The solutions also walked more slowly and showed higher evaluation-window mean hip-to-knee loading. Sagittal mean joint angles changed modestly, pelvis-orientation range decreased, and activation shifted from vasti toward gluteus maximus and iliopsoas. C2 retained substantial threshold exceedance, indicating that the 2-BW soft target was not achieved under the concurrent objective terms. These findings describe selected solutions of one model and terrain traversal, not a population-average clinical response.

The results are consistent with the general concept that pain-related motor adaptations can redistribute mechanical demand (4). In this simulation, knee peak and impulse reductions co-occurred with timing, speed, and muscle-coordination changes while mean sagittal joint angles changed relatively little. The pelvis coordinate was analyzed only as a model kinematic variable; no lumbar strategy can be inferred.

The present results should not be interpreted as experimental validation of a real-world pain-avoidance strategy. They show how one model-controller-objective combination resolved competing soft penalties. The selected speeds, loads, and redistribution ratios are conditional on the inherited weights, simplified contact model, and deterministic terrain sequence.

### 4.2 Relation to experimental gait adaptations in knee osteoarthritis

Experimental knee-osteoarthritis gait studies commonly report slower walking, reduced knee flexion or extensor moments, and compensatory changes at adjacent joints (15–18). The retained simulations reproduced a qualitatively similar direction of change in speed, vasti activation, and hip demand. However, this qualitative agreement is not validation of the absolute joint reaction forces. Because speed itself changes kinematics and ground reaction forces (19), the convergence of C4-C2 toward the minimum-speed term should be viewed as part of the optimized trade-off, not a disease-specific prediction.

### 4.3 Feasibility of strict knee-load limiting under uneven-terrain demands

Instrumented knee-implant studies generally report resultant forces of approximately 2.2–3.5 BW for many daily activities, including walking (20, 21). The C0 maxima of approximately 8.3 BW are therefore not physiologically validated and exceed the usual experimental range. Our localization analysis showed that both occurred during early stance after initialization. The left peak occurred in a stance confined to the −4° tile, whereas the right peak occurred 0.047 m beyond the −4° to −2° boundary within a transition stance. Thus, terrain-transition dynamics may have contributed to the right peak but cannot explain the left peak. The likely contributors include heel-strike dynamics from the compliant two-sphere-per-foot contact, the simplified controller/objective, and the fact that the reported variable is a resultant pin-joint reaction rather than tibiofemoral contact force. The load-limited maxima of 3.6–4.5 BW are closer to, but still not directly interchangeable with, implant contact-force measurements. Accordingly, relative reductions are more defensible than claims about physiological absolute loads.

Terrain-specific evaluation replaced the earlier speculation about slope. The C0 left peak occurred during a stance confined to the −4° tile, whereas the right peak occurred on the −2° tile immediately after crossing from the −4° tile. In contrast, the C4 maxima occurred on −2° and −8° tiles, and the C3-C2 maxima occurred on −4° and −2° tiles. Thus, neither the steepest decline, a single terrain class, nor terrain transition alone explained all residual peaks. Each tile contributed at most a small number of stances, precluding inferential comparisons among slopes; the step-level results should be interpreted descriptively.

Because there was no level-ground comparator, repeated terrain sequence, or alternative slope order, the findings are framed as behavior during this specific uneven-terrain stress test rather than effects caused by uneven terrain itself. Replicated terrain sequences and level walking are needed to test terrain-specific generalizability.

### 4.4 Implications, limitations, and future work

The stance-level analysis provides a more interpretable summary of cumulative knee demand than the whole-window mean: bilateral-average knee impulse decreased by 13%–23%, whereas average loading rate did not change monotonically. Because the outcome is a resultant pin-joint reaction rather than a compartmental contact force and no clinical sample or outcome was studied, these findings do not support a minimal clinically important change or a specific intervention recommendation. The increased hip-to-knee mean ratio should therefore be regarded as a hypothesis-generating signal within the simulation.

Interpretation is constrained by the sagittal-plane H0918v3 model, including a rigid head-arms-trunk segment, absent lumbar, thoracic, and metatarsophalangeal degrees of freedom, sparse musculature, simplified foot-ground contact, and pin-joint knees without compartmental load sharing. Pelvis tilt consequently describes whole-segment orientation rather than lumbar motion, and the resultant knee reaction force should not be equated with cartilage contact force.

Results are also conditional on the H0918RS2v3 reflex controller, inherited objective weights, default_material friction parameters (not varied), deterministic ten-tile sequence, and the retained lowest-objective trajectory. The objective distributions showed that 5–17 runs per condition were within 10% of the selected score, but nonselected trajectories were not replayed; thus, the selected behavior cannot be assumed representative of near-optimal solutions. No direct baseline validation, level-ground comparator, replicated terrain sequence, or physiological pain model was available. Accordingly, the findings describe feasible behavior under the specified architecture and evaluation window and should not be generalized to other terrain sequences, material pairs, patient populations, or clinical interventions. Future work should combine baseline validation with replay of near-optimal runs, objective-weight and contact-parameter sensitivity analyses, replicated level and uneven terrain, and three-dimensional models with compartment-capable knees.

In conclusion, within the retained solutions of this sagittal-plane model, controller, objective weighting, and deterministic terrain traversal, bilateral soft knee-load limiting reduced stance-phase knee reaction-force maxima and impulses while slower walking and higher time-averaged hip-to-knee loading emerged. The 2-BW target remained substantially exceeded, and loading rate did not improve monotonically. These scenario-specific findings motivate validation against experimental loads, replay of multiple near-optimal trajectories, weight sensitivity analysis, level and replicated terrain conditions, and three-dimensional compartment-capable models.

## Data Availability

The original contributions presented in the study are included in the article/Supplementary Material, further inquiries can be directed to the corresponding author.
