# Supplementary material for: Bilateral knee-load limiting during uneven-terrain walking induces hip-dominant load redistribution in predictive gait simulations
Source: Front Med (Lausanne). 2026 Sep 28;13:1912600. doi: 10.3389/fmed.2026.1912600 (PMC13654721; doi:10.3389/fmed.2026.1912600)
Supplement: Supplementary file 1 [file Supplementaryfile1.docx]

Supplementary Materials

# 1 Supplementary Figures

**Supplementary Figure 1.** Sagittal-plane kinematic trajectories across conditions for the left side. Raw SCONE radian coordinates are shown without sign reversal. Hip_flexion is positive for flexion, knee_angle becomes more negative with knee flexion, ankle_angle becomes more negative with plantarflexion, and pelvis_tilt is the global pelvis-orientation coordinate. The sign-reversed pelvis summary in the main manuscript does not represent lumbar motion. Solid lines and bands show within-simulation means and SDs across mechanically distinct steps and are descriptive rather than repeated-trial estimates of one homogeneous task.

**Supplementary Figure 2.** Muscle-activation profiles (0-1) for the left side. Muscles and plotting format are identical to Figure 4. Solid lines and bands show within-simulation means and SDs across mechanically distinct steps; background shading indicates controller gait subphases.

# 2 Supplementary Tables and Data

**Supplementary Table S1.** Terrain and timing of the maximum knee resultant joint reaction force in each retained condition and limb.

| **Condition** | **Limb** | **Analyzed stance** | **Full-sim stance** | **Heel strike (s)** | **Peak time (s)** | **Peak (BW)** | **Peak tile / stance terrain** | **State** |
| --- | --- | --- | --- | --- | --- | --- | --- | --- |
| C0 | Left | 5 | 6 | 6.47 | 6.60 | 8.35 | Peak: Tile 9 / -4° Stance: Tile 9 (-4°) | Early stance |
| C0 | Right | 5 | 6 | 7.10 | 7.24 | 8.25 | Peak: Tile 10 / -2° Stance: Tile 9 (-4°) → Tile 10 (-2°) | Early stance |
| C2 | Left | 6 | 7 | 8.91 | 8.94 | 3.60 | Peak: Tile 9 / -4° Stance: Tile 9 (-4°) | Early stance |
| C2 | Right | 7 | 7 | 9.52 | 9.55 | 3.73 | Peak: Tile 10 / -2° Stance: Tile 10 (-2°) | Early stance |
| C3 | Left | 6 | 7 | 8.27 | 8.30 | 3.63 | Peak: Tile 9 / -4° Stance: Tile 9 (-4°) | Early stance |
| C3 | Right | 7 | 7 | 8.85 | 8.88 | 3.71 | Peak: Tile 10 / -2° Stance: Tile 10 (-2°) | Early stance |
| C4 | Left | 7 | 8 | 9.38 | 9.40 | 4.51 | Peak: Tile 10 / -2° Stance: Tile 10 (-2°) | Early stance |
| C4 | Right | 6 | 6 | 7.53 | 7.89 | 4.34 | Peak: Tile 8 / -8° Stance: Tile 8 (-8°) | Early stance |

**Supplementary Table S2.** Complete-stance knee reaction-force outcomes by condition and limb.

| **Condition** | **Limb** | **n** | **Mean peak (BW)** | **Maximum (BW)** | **Mean impulse (BW·s)** | **Mean average loading rate (BW/s)** |
| --- | --- | --- | --- | --- | --- | --- |
| C0 | Left | 8 | 4.88 | 8.35 | 2.14 | 30.03 |
| C0 | Right | 8 | 4.23 | 8.25 | 2.00 | 17.20 |
| C2 | Left | 7 | 2.71 | 3.60 | 1.58 | 28.68 |
| C2 | Right | 8 | 2.80 | 3.73 | 1.59 | 28.41 |
| C3 | Left | 8 | 2.96 | 3.63 | 1.65 | 17.40 |
| C3 | Right | 8 | 3.02 | 3.71 | 1.69 | 23.01 |
| C4 | Left | 8 | 3.45 | 4.51 | 1.79 | 35.80 |
| C4 | Right | 8 | 3.37 | 4.34 | 1.82 | 24.28 |

**Supplementary Table S3.** Objective-score distribution across 24 independent optimizations.

| **Condition** | **Selected** | **Median** | **Range** | **Within 10%, n** | **Within 25%, n** | **Runs, n** |
| --- | --- | --- | --- | --- | --- | --- |
| C0 | 1.470 | 1.657 | 1.470-1.991 | 6 | 21 | 24 |
| C4 | 1.778 | 2.276 | 1.778-7.146 | 5 | 12 | 24 |
| C3 | 1.901 | 2.144 | 1.901-2.913 | 8 | 22 | 24 |
| C2 | 2.866 | 3.124 | 2.866-3.372 | 17 | 24 | 24 |

**Supplementary Table S4.** Reconstructed weighted objective-component contributions for the retained trajectories.

| **Condition** | **Total** | **Gait*** | **Effort** | **Activation** | **DoF limits** | **GRF** | **Knee cap** |
| --- | --- | --- | --- | --- | --- | --- | --- |
| C0 | 1.470 | 0.092 | 0.059 | 0.926 | 0.095 | 0.298 | 0.000 |
| C4 | 1.778 | 0.169 | 0.072 | 1.014 | 0.116 | 0.395 | 0.011 |
| C3 | 1.901 | 0.137 | 0.070 | 1.036 | 0.147 | 0.474 | 0.037 |
| C2 | 2.866 | 0.154 | 0.077 | 1.160 | 0.177 | 0.528 | 0.771 |

*The gait contribution is the residual required to reproduce the selected total objective, which was stored to three decimals in the result filename. Other terms were reconstructed from stored per-frame penalty channels and configured weights. Supplementary Data S1 provides all 63 complete stances, including timing, contacted terrain, peak location, impulse, and average loading rate.
